# Infectious Bronchitis Coronavirus: Genome Evolution in Vaccinated and Non-Vaccinated SPF Chickens

**DOI:** 10.3390/v14071392

**Published:** 2022-06-25

**Authors:** Alexandre Flageul, Chantal Allée, Céline Courtillon, Véronique Béven, Hélène Quenault, Yannick Blanchard, Michel Amelot, David Courtois, Sjaak De Wit, Nicolas Eterradossi, Béatrice Grasland, Paul A. Brown

**Affiliations:** 1 Virology, Immunology, Parasitology, Aviaire et Cunicole, French Agency for Food, Environmental and Occupational Health & Safety (ANSES), Laboratoire de Ploufragan-Plouzané-Niort, Université de Rennes 1, 22440 Ploufragan, France; al.flageul@laposte.net (A.F.); chantal.allee@anses.fr (C.A.); celine.courtillon@anses.fr (C.C.); nicolas.eterradossi@anses.fr (N.E.); beatrice.grasland@anses.fr (B.G.); 2 Génétique Virale et Biosécurité, French Agency for Food, Environmental and Occupational Health & Safety (ANSES), Laboratoire de Ploufragan-Plouzané-Niort, Université de Rennes 1, 22440 Ploufragan, France; veronique.beven@anses.fr (V.B.); helene.quenault@anses.fr (H.Q.); yannick.blanchard@anses.fr (Y.B.); 3 Service d’Elevage et d’Expérimentation Avicole et Cunicole, French Agency for Food, Environmental and Occupational Health & Safety (ANSES), Laboratoire de Ploufragan-Plouzané-Niort, Université de Rennes 1, 22440 Ploufragan, France; michel.amelot@anses.fr (M.A.); david.courtois@anses.fr (D.C.); 4 Royal GD, Department of Farm Animal Health, Faculty of Veterinary Medicine, Utrecht University, 7418 EZ Deventer, The Netherlands; j.d.wit@gddiergezondheid.nl

**Keywords:** infectious bronchitis, coronavirus, vaccination, evolution, variants

## Abstract

Infectious Bronchitis virus (IBV) continues to cause significant economic losses for the chicken industry despite the use of many live IBV vaccines around the world. Several authors have suggested that vaccine-induced partial protection may contribute to the emergence of new IBV strains. In order to study this hypothesis, three passages of a challenge IBV were made in SPF chickens sham inoculated or vaccinated at day of age using a live vaccine heterologous to the challenge virus. All birds that were challenged with vaccine heterologous virus were positive for viral RNA. NGS analysis of viral RNA in the unvaccinated group showed a rapid selection of seven genetic variants, finally modifying the consensus genome of the viral population. Among them, five were non-synonymous, modifying one position in NSP 8, one in NSP 13, and three in the Spike protein. In the vaccinated group, one genetic variant was selected over the three passages. This synonymous modification was absent from the unvaccinated group. Under these conditions, the genome population of an IBV challenge virus evolved rapidly in both heterologous vaccinated and non-vaccinated birds, while the genetic changes that were selected and the locations of these were very different between the two groups.

## 1. Introduction

Viruses in the Coronaviridae family have a large single-stranded positive-sense RNA genome. Within this family, two sub-families have been described, namely, the Letovirinae and the Orthocoronavirinae. In the latter, four genera have been established, including the Gammacoronavirus genus, of which the infectious bronchitis virus (IBV) is the prototype virus [1].

IBV was first isolated in the United States of America (USA) in 1933 by Bushnell and Brandly from chickens with Infectious Bronchitis (IB), a respiratory disease of chickens initially described in 1931 by Schalk and Hawn [2]. Following initial isolation, many different genotypes and antigenic types have been discovered around the world [3,4], several of which produce clinical manifestations other than or in addition to respiratory symptoms, including reproductive problems such as egg-drops and false layer syndrome or nephritis. All continue to cause serious economic losses for the poultry industry [5]. To date, no anti-viral treatment exists for IBV; infection is controlled through vaccination with live-attenuated and inactivated viruses and by strict attention to hygiene, such as complete cleaning and disinfection of animal houses and using all-in all-out strategies.

Live-attenuated vaccines are the primary source of protection in young poultry and inactivated vaccines serve as a support to boost the immune response in long-living birds. However, due to the diversity of IBV strains around the world, no single live-attenuated vaccine confers protection against all strains. This has led to the development of an important number of live-attenuated vaccines derived from different IBV strains and their use in many different vaccination programs. For instance, in Poland, over a period of twenty months more than 70 vaccination protocols were inventoried [6]. The heavy use of live-attenuated vaccines has been suggested as one potential factor contributing to the emergence of new IBV strains, either through the introduction of IBV vaccine strains that were not previously indigenous to the geographic region, through increased chances of recombination, or through pressure/selection imposed by pre-existing immunity [7,8] offering only partial protection. These new strains are viruses that carry genetic modifications compared to a parental genome reference, and are often referred to as “variants”. Declarations of genetic variants most often refer to the majority sequence of a virus population, and for IBV it has been suggested that this majority should demonstrate a nucleotide difference of >13% in the S1 region of the S ORF in order to warrant identification as a variant [4]. These parameters were suggested to reduce the number of declarations and to take into account the fact that most variants of importance have a reasonable number of nucleotide changes. However, variants with a high number of genetic differences throughout the genome do not necessarily correlate to a virus with important phenotypic changes; this depends on a number of other factors, such as whether the changes occur in regions of importance for RNA structure or whether they result in a change of encoded amino acid, which can affect the tertiary structure of a protein. Thus, literally speaking, a genetic variant within a viral genome population may possess one or a number of nucleotide changes compared to the majority or consensus sequence which, if selected over a certain time period due to a particular pressure, can produce a new majority sequence with few changes or even a new majority sequence with numerous changes. Both types of genetic variants have the potential to generate viruses with different phenotypic traits. 

In order to address the question of whether a pre-existing immunity raised against a live-attenuated vaccine of a particular IBV serotype encountering heterologous viral infection can result in a difference in the evolution of the infecting viral genome population compared to subjects without pre-existing immunity, the following experiment was conceived. Chickens were vaccinated at a day old with a Massachussetts serotype [9], namely, live-attenuated IBV vaccine H120 (the most frequently used IBV vaccine worldwide) or with a placebo, then subjected to three passages of H120-heterologous IBV challenge virus D388 (QX genotype) [10]. In parallel, a group of the same vaccinated chickens were challenged with H120-homologous IBV strain M41 in order to control the experimental design and the efficacy of the vaccine-induced immunity in blocking homologous virus replication. The H120 vaccine was used

Viral RNAs of the heterologous challenge virus replicating in the tracheas and kidneys of vaccinated birds and in those given the placebo were sequenced using Next Generation Sequencing (NGS) to compare their evolution.

## 2. Materials and Methods

### 2.1. IBV Vaccine and Challenge Viruses

Lyophilized H120 vaccine (BIORAL) was re-suspended in 100 mL of stabilizing buffer (STAB, composed of Saccaharose [20 g/L], KH2PO4 [0.52 g/L], NaHPO412H2O [2.58 g/L],]) according to the manufacturer’s recommendations. 

Lyophilized D388 [10] was re-suspended in 1 mL of STAB. Re-suspended IBV-D388 and a virus stock of IBV-Mass41 (Salisbury, MA, USA, 1971) were titrated in eggs [11] using the Reed and Muench method [12]. D388 was diluted in STAB to adjust the viral titer to 10^5^ egg infectious dose (EID) 50/mL, and constituted the D388 inoculum. Mass41 titer was 10^5.33^ EID50/mL and was not diluted. 

### 2.2. Preparation of Embryonic Chicken Kidney Cells (eCK)

Chicken embryos of 19 days of age (d.o.a.) were used. Kidneys were collected and washed three times with Phosphate Buffer Saline (PBS) at 37 °C for three minutes in an Erlenmeyer flask with gentle agitation. Cells were then dissociated by two successive incubation steps of one and three minutes at 37 °C with an excess of fresh and warm PBS supplemented with trypsin (0.05%), ethylenediaminetetraacetic acid (EDTA, 0.1%), penicillin (100 units (U)/mL), and streptomycin (100 µg/mL). Foetal calf serum was added (10% final) to stop the enzyme activity and the suspension was centrifuged at 375× *g*, for 5 min at room temperature. Cells were re-suspended in Glasgow Minimum Essential Medium with glutamine plus tryptose phosphate (30 mg/mL), penicillin (100 U/mL), and streptomycin (100 µg/mL), then filtered over six layers of sterile gauze band. Filtered cells were added to 96-well plates (Nunclon™ Delta surface, Thermo Scientific, Courtaboeuf, France) at a concentration of 5.10^5^ cells per milliliter and the plates were incubated at 37 °C with 1.5% CO_2_.

### 2.3. Experimental Design

All experiments were performed in accordance with EU and French regulations on animal welfare in experiments. The protocol (Reference APAFIS#5178-20l6041118128688 v5) was approved by the ANSES/ENVA/UPEC Ethical Committee Registry No. 16 and by the French ministry of Higher Education, Research and Innovation.

Two experimental trials were carried out and codified, as follows: trial H120-D388 (vaccination with H120 and challenge with heterologous D388) and trial H120-Mass41 (vaccination with H120 and challenge with homologous Mass41).

#### 2.3.1. Trial H120-D388

Thirty specific pathogen-free (SPF) White Leghorn chickens, Anses Ploufragan-Plouzané-Niort laboratory, Ploufragan, France, of one d.o.a. were separated into two groups of fifteen birds; each group was placed in a rearing isolator in the Anses Biosafety Level 2 facilities. The first group was vaccinated at one d.o.a. by eye drop with one dose (60 µL) of H120 live-attenuated vaccine (vaccinated birds (VB)). The second group was sham-inoculated in the same way with STAB medium (non-vaccinated birds (NVB)). At 24 d.o.a., blood was collected from all birds to check their IBV immune status, as reflected by the presence of antibodies against IBV (see below “IBV Enzyme-Linked Immuno-Sorbent Assays”). For VB and NVB, five animals of 24 d.o.a. were moved to a new isolator and then challenged by eye drop with 10^4^ EID_50_ of D388 inoculum. At 29 d.o.a. (five days post-challenge), challenged animals were humanely euthanized. Blood, tracheal, and kidney tissues were then collected from these birds. Tracheal and kidney tissues were individually homogenized in STAB medium (1 g/mL) and centrifuged (1000× *g*, 10 min, 4 °C). Supernatants were collected and considered as the viral stocks of the first passage of the challenge D388 in VB and in NVB. Viral stocks were labelled with the bird number followed by “D388pass1TrVB” or “D388pass1TrNVB” for the tracheas and by “D388pass1KdVB” or “D388pass1KdNVB” for the kidneys. Two hundred microliters from each of the five D388pass1TrVB stocks and of the five D388pass1KdVB stocks were mixed together to create a pooled virus stock, “D388pass1TrKdVB”. The same procedure was performed on viral stocks from NVB (pooled virus stock “D388pass1TrKdNVB”). All individual viral stocks were then stored at −80 °C for later analysis by NGS.

At 29 d.o.a., two new groups of five VB and five NVB were moved to new isolators and challenged with D388pass1TrKdVB or D388pass1TrKdNVB, respectively. After challenge, the process described above for passage one was repeated and was considered as the result of the second passage. The resulting individual viral stocks were labelled by replacing “pass1” with “pass2”.

At 36 d.o.a., the final groups of five VB and five NVB were challenged with the pooled virus stocks D388pass2TrKdVB or D388pass2TrKdNVB, respectively, in the rearing isolators. Following challenge, the process described above for passage one was repeated and was considered as the result of the third passage. The resulting viral stocks were labelled by replacing “pass1” with “pass3”.

Blood samples were taken at each passage immediately before challenge to check IBV immune status, and all viral stocks were tested for the presence and quantity of viral RNA using a previously described pan-avian coronavirus, RT-qPCR [13]. Clinical signs (sneezing, rales, prostrations [14]) were monitored for the duration of the trial and macroscopic lesions were noted during necropsies (for collection of tracheas and kidneys).

#### 2.3.2. Trial H120-Mass41

The only change from the trial described above was the use of Mass41 as the challenge virus. Virus samples were coded in the same way. The only difference was the replacement of D388 by Mass41; for example, the stock equivalent to D388pass1TrVB in trial H120-D388 was labelled Mass41pass1TrVB in this trial.

### 2.4. Measuring Anti-IBV Antibodies by Enzyme-Linked Immuno-Sorbent Assays (ELISA)

Blood samples were centrifuged (1160× *g*, 15 min, 4 °C) and sera were tested with a commercial kit, IBV CK119 (BioChek, Reeuwijk, The Netherlands), according to the manufacturer’s instructions. The results were interpreted as recommended, with a positivity threshold of absorbance of 0.2.

### 2.5. Preparation of Viral Sample for NGS

All samples from the H120-D388 trial and the D388 inoculum were passaged one time in embryonated SPF chicken eggs (eggP1) to optimize samples for NGS. This system of optimization was chosen because it has been promoted as an efficient method for producing high levels of NGS data from IBV isolates [15]. The D388 inoculum was processed directly by RNA extraction and NGS without egg passage as well, in order to allow the effects of one egg passage on a virus population to be assessed.

For egg passaged viruses, individual samples were diluted (1/10) in PBS supplemented with penicillin (10 U/µL), streptomycin (10 µg/µL), and amphotericin B (250 µg/µL) and incubated for one hour at 4 °C. These were then inoculated into the allantoic cavity of nine-day-old embryonated SPF chicken eggs. Eggs were incubated for six days at 37 °C and candled every day to check for embryo mortality. Chicken embryos that died during the first 24 h of incubation were discarded. At the end of the incubation period, or for embryos that died later than 24 h post inoculation, allantoic fluids were collected as previously described [16] then centrifuged (1160× *g*, 15 min, 4 °C) to constitute the viral stocks for NGS analysis.

### 2.6. RNA Extraction

RNA was extracted from the different virus samples prepared above with the KingFisher™ Flex Purification System (Thermo Scientific Courtaboeuf, France) using magnetic beads, washing, and elution buffers from a NucleoMag Vet kit. Briefly, 200 µL of samples were lysed with 300 µL of RLT buffer supplemented with 3 µL of β-mercaptoethanol and 16 µg of glycogen. After the lysis step, 20 µL of magnetic beads and 400 µL of isopropanol were added for RNA precipitation. Three washing steps were performed: two using washing buffers and one using 80% ethanol. Finally, RNA was eluted using 100 µL of elution buffer.

### 2.7. Illumina Sequencing and NGS Data Processing

RNA libraries were prepared using the NEBNext Ultra II Directional RNA library. Briefly, ribosomal RNA was removed with RNAse H and remaining RNA was converted into double-stranded DNA (dsDNA) by random reverse transcription and second-strand DNA synthesis. Then, sequencing adaptors were ligated to dsDNA and a short PCR enrichment was performed before sequencing. NGS was performed using NextSeq Illumina technology. NGS data were processed twice with the recently developed Viral Variant Visualizer (VVV) pipeline [17] to assess IBV variants. VVV was chosen due to its simple graphical outputs that simplify these types of analysis for non-bioinformaticians. The first analysis involved all the reads in the NGS file, while the second analysis removed reads considered as duplicates (identical reads). For the reasons previously defined [17], only genetic variants detected in both analyses and present in the viral population above a threshold value of 7% were analysed. Data were considered robust when the median coverage depth was greater than 500 and when a minimum of 200 reads was present at all locations. Other data were annotated as “low coverage”.

### 2.8. In Silico and In Vitro Analysis of Variant Viruses with Modified Spike Protein Sequences

PSI-blast analysis [18,19], using defaults parameters and restricted to the avian coronaviruses, was performed to search for homologous proteins in the database. Variant viruses with modified S proteins were tested in virus neutralization assays as described below.

#### Viral Neutralization and Immunofluorescence Assays

EggP1-D388 inoculum (one D388pass3TrNVB, one D388pass3KdNVB, one D388pass3TrVB, and one D388pass3KdVB) was used for constant serum-virus diluting viral neutralization experiments in eCK cells. Viruses were serially diluted from 10^−1^ to 10^−5^ and distributed into two duplicate 96-well plates (plate 1 and 2) using 75 µL of dilution per well. Then, 75 µL of BHK medium was added to each well of plate 1 and incubated at room temperature for one hour, and 75 µL of anti-D388 polyclonal serum (diluted 1/27 in BHK medium) was added to each well of plate 2, then incubated at room temperature for one hour. Following the one-hour incubation period, 125 µL of eCK cells were added to the wells of the two plates. Both plates were then incubated for two days at 37 °C with 1% CO_2_. After the two-day incubation period, the supernatants were removed and the cells were washed with PBS. Cells were fixed for 20 min at −20 °C with a mix of ice-cold ethanol/acetone (ratio 1:1). Ethanol/acetone was removed and cells were dried before storage at −20 °C.

For immunofluorescence assay, plates were thawed, defrosted, washed with PBS, and incubated for one hour at room temperature with a mouse monoclonal antibody targeting the IBV Membrane protein (mAb-IBV-25.1, Wageningen Bioveterinary Research, diluted 1/200 in PBS, previously known to cross-react with a range of avian coronaviruses) (data not shown). After three washes with PBS, cells were incubated with AlexFluor 488 goat anti-mouse IgG (H+L) (diluted 1/800 in PBS) for 45 min at room temperature. Cells were washed and stored in PBS at 4 °C. Immunofluorescence was observed using FITC filter on an Olympus IX71 microscope with CoolLED pE-300 as source of light.

### 2.9. Statistical Analysis

The relative mean concentrations of viral RNA in vaccinated and unvaccinated birds at all passages of the H120-D388 trial were compared using the Wilcoxon test from the R stats package v4.0.3. (R Core Team. R: A Language and Environment for Statistical Computing. 4.0.3) due to the non-normal distribution of the values. 

## 3. Results

### 3.1. H120-D388 Trial

Prior to vaccine/placebo inoculation, all birds were seronegative for IBV. In the NVB group, birds were all seronegative prior to the different challenges (Table 1). Five days after the initial D388 challenge, one bird had died. At necropsy, this bird had hypertrophied kidneys. During passage 2, another bird showed signs of prostration in the evening of the fourth day after challenge. The following morning this bird had died’ at necropsy, it had a haemorrhagic liver, hypertrophied kidneys, and petechial trachea. Clinical signs were not observed for any other animals throughout the duration of the trial. However, at necropsy all birds in passage 3 had hypertrophied kidneys, while the birds in passages 1 and 2 did not. Viral RNA was detected in the tracheas (mean 10^5.69^ copy/µL SD-0.94) and kidneys (mean 10^5.72^ copy/µL SD-0.96) of all NVB birds in all passages, with the exception of one bird (passage 3) with a positive kidney sample and negative tracheal sample (Table 1).

**Figure 1:**
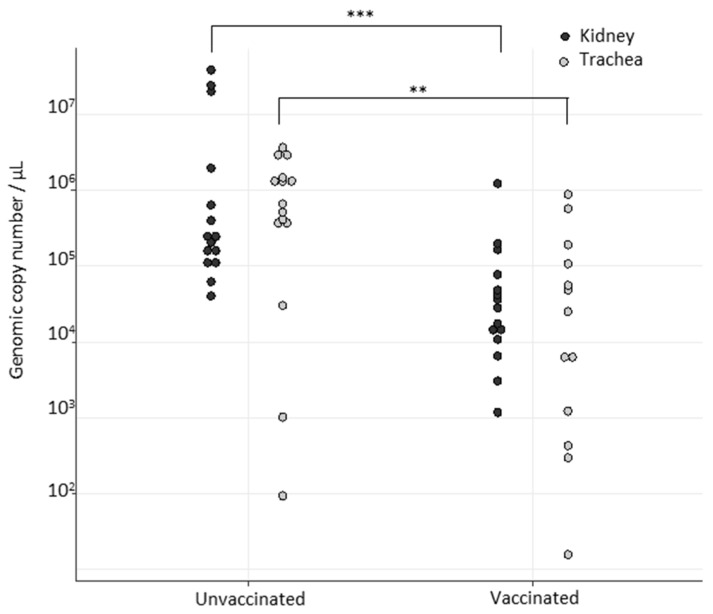
Comparison of relative mean of D388 viral RNA in both tracheas and kidneys of vaccinated and unvaccinated subjects. Statistical tests (comparison of mean of two independent samples) were performed using Wilcoxon test. ** *p*-value below 0.001; *** *p*-value below 0.0001.

In the VB group, two out of five, two out of five, and five out of five birds were seropositive on the day of challenge in passage 1, 2, and 3, respectively (Table 1). No clinical signs or gross lesions were observed on any of the animals throughout the duration of the trial, except for one bird in passage 2 that had hypertrophied kidneys and petechial trachea. This bird was seronegative before challenge. Viral RNA was detected in the kidneys of all birds in all passages (mean 10^4.44^ copy/µL SD-0.75), while viral RNA was detected in the tracheas of three out of five (mean 10^3.48^ copy/µL SD-1.11), five out of five (mean 10^4.70^ copy/µL SD-0.94), and three out of five birds (mean 10^3.77^ copy/µL SD-1.89) in passage 1, 2, and 3, respectively (Table 1). Viral genomic loads in the tracheas and kidneys were lower than those found in the same tissues of the NVB animals (Figure 1). 

### 3.2. Comparison of D388 Genome Evolution in VB and NVB

#### 3.2.1. Sequencing of eggP1 D388 Inoculum

**Figure 2:**
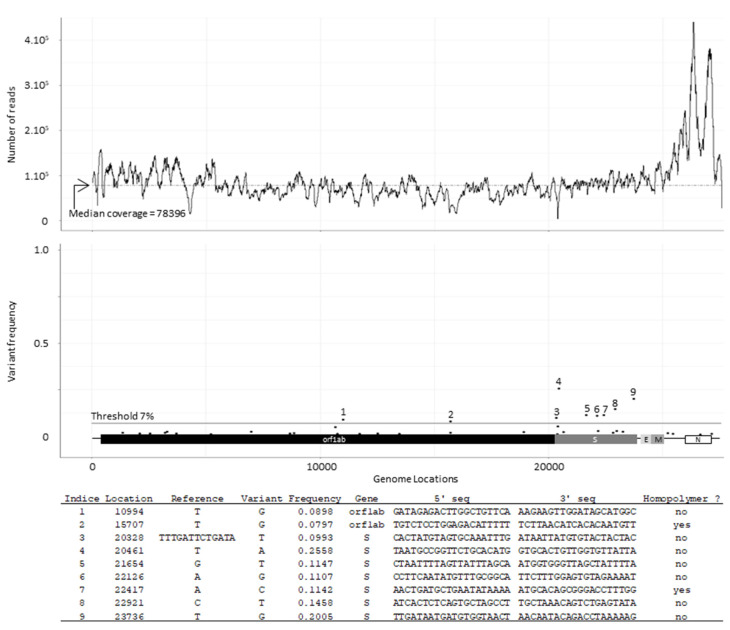
Genetic variant map of the EggP1 D388 inoculum. EggP1 D388 inoculum was deep sequenced and NGS data were processed through VVV. The upper graphic shows genome coverage, While the middle graphic presents detected genetic variants over the viral genome; numbers = variants above the threshold. The table provides the details of the detected genetic variants (1–9).

The consensus sequence of eggP1 D388 inoculum was established from NGS data, generating a median coverage depth of 78,396 (Figure 2) and a coverage depth of 497 when identical reads were removed. Both sets of data resulted in the detection of nine viral genetic variants, with percentage of presence comprising between 8 and 26% (Figure 2). Among them, two were in the orf1ab at positions 10994 and 15707 (coding sequences of NSP8 and NSP13 respectively) and the other seven were detected in the S coding sequence at positions 20328, 20461, 21654, 22126, 22417, 22921, and 23736. The two variants in orf1ab and those at positions 21654, 22417, 22921, and 23736 in the S coding sequence were found in the D388 inoculum before egg passage as well. EggP1 D388 and D388 shared the same consensus sequence. 

#### 3.2.2. Data Processing and Compilation

**Figure 3:**
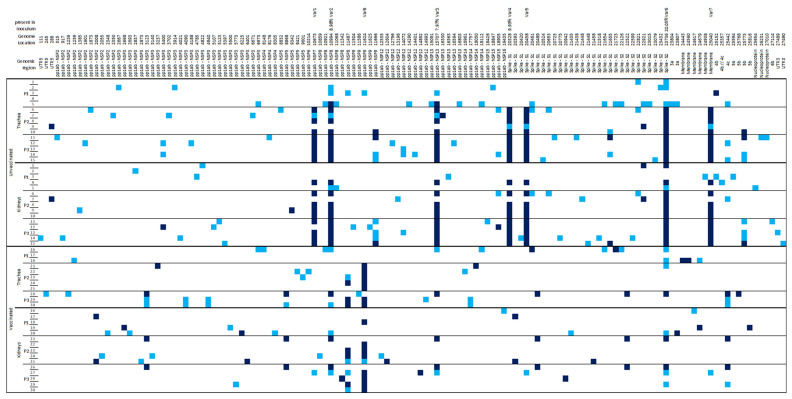
D388 variants detected in tracheal and kidney tissues of VB and NVB in passage 1, 2, and 3. Variants are indicated by light blue squares when they were detected in less than 50% of the population and by dark blue squares when they were detected in 50% or above. Variants for each individual at each passage are presented by line. Genomic regions and with genome locations for the variants are indicated above the graphic. Percentages of Var1 to Var8 in the viral population of the inoculum are shown next to the genome location when detected.

NGS data obtained from RNA extraction of the different viral samples and the D388 inoculum were processed through the VVV pipeline [16]. VVV was programmed to compare data from viral samples after one passage in eggs to the consensus sequence of the D388 inoculum after one passage in eggs. All detected variants were compiled into a single graphic, which is shown in Figure 3. Each variant is indicated by a blue square, which is a lighter blue colour if the variant frequency was less than 50% of the total sequences at that position or a darker blue colour if the variant was above the threshold of 50% of the total sequences; in other words, the colour indicates a consensus level change.

For all data, analysis involving all reads resulted in median coverage depths comprising between 5136 and 19647, except for data from the RNA extraction of 17-D388pass1TrVB and the extraction of 29-D388pass3TrVB, which were 105 and 124, respectively. 

#### 3.2.3. Evolution of D388 Genome Population in NVB

A total of 58 and 50 variants were detected throughout the three passages in the trachea and the kidney, respectively. Seven of these variants (Var1 to 7) were selected over passage in both tissues, and by the third passage had arrived at the consensus level in all birds except one, which uniquely displayed the change in the trachea (Figure 3). Four variants out of the seven were detected in the genome population of the EggP1 D388 inoculum: Var2 (composing initially 9% of the viral population), Var3 (8%), Var4 (10%), and Var6 (20%).

#### 3.2.4. Evolution of D388 Genome Population in VB

A total of 36 and 39 variants were detected throughout the three passages in the trachea and kidney, respectively. One of these variants (Var8) was selected over passage in both tissues (Figure 3). Var8 was not detected in the genome population of EggP1 D388. 

### 3.3. Predicted Protein Impact of the Non-Synonymous Selected Mutations

Of the eight variants described above, Var2, 3, 4, 5, and 6 (NVB) were predicted to modify the amino acid (aa) sequence. Several of these were the result of an impact on a particular codon, while others were the result of deletion modifications.

Var2 (nucleotide (nt) mutation T-10994-G) modified the aa sequence (H-3520-Q) of PP1ab in the NSP8 region. Var2 replaced one polar positively-charged aa with one polar uncharged aa. After two iterations of PSI-blast, 556 sequences of avian Gammacoronavirus NSP8 were collected; within these sequences, the Glutamine of the variant was found in 555 sequences and the Histidine was not present (data not shown).

Var3 (nt mutation T-15707-G) modified the aa sequence (F-5092-V) of PP1ab in the NSP13 region. Var3 replaced one bulky hydrophobic aa with another of smaller size. After two iterations of PSI-blast, 438 sequences of avian Gammacoronavirus NSP13 were collected; within these sequences, the Valine of the variant viruses was found in 413 sequences and Phenylalanine was present in four sequences (data not shown).

Investigation of Var4 and Var5 was conducted together, as the changes observed in these variants took place in the S1 region of the Spike protein (upstream and in the middle of the hypervariable region 1, respectively). Var4 had the deletion TTGATTCTGATA (20328–20340), which resulted in the deletion of four aa (DSDN at position 21–24) and a change F-to-Y at position 20. Var5 (nt mutation ATGT-20458-GTGA) modified the aa sequence (HV-64-RE), replacing one positively charged aa and one hydrophobic aa with two positively charged aa. Because of these substantial modifications in the Spike protein, it was hypothesised that they may produce viruses that could not be neutralized by antibodies raised against the D388 inoculum, and these viruses were included in subsequent VN tests. Var6 (nt mutation T-23736-G) replaced the stop codon of the Spike protein with a glutamic acid, resulting in an extension of the Spike protein by nine aa (stop-1157-EQYRPKKSVstop). This extended version of the Spike protein had previously been deposited in GenBank databases for most IBV sequences (444 out of 483 analysed S gene of IBV).

### 3.4. Virus Neutralization of Viral Stocks Containing Vars 4 and 5 with Anti-D388 Polyclonal Serum

D388 was able to infect eCK cells in the absence of antibodies raised against the D388 inoculum, yet was not able to infect eCK cells after prior adsorption with the anti-D388 polyclonal serum. The Var4, Var5, and Var8 viruses were not able to infect eCK cells under any condition, and thus neutralization studies could not be performed.

### 3.5. H120-Mass41 Clinical Results

Prior to vaccine/placebo inoculation, all birds were seronegative for IBV. In the NVB group, all birds were seronegative prior to the different challenges (Table 2). Throughout the trial, NVB challenged with Mass41 remained healthy and did not display any notable clinical signs. No gross lesions were observed during necropsies of any birds. Five days after challenge, viral RNA (mean 10^6.87^ copy/µL SD-0.47) was detected in the trachea of all birds of all passages, while viral RNA was detected in the kidneys of two out of five (mean 10^4.00^ copy/µL SD-2.15), two out of five (mean 10^2.95^ copy/µL SD-0.97), and four out of five birds (mean 10^5.77^ copy/µL SD-0.55) in passage 1, 2, and 3 respectively.

In the VB group, two birds died before challenge. Two out of four, four out of five, and three out of four were seropositive on the day of challenge in passage 1, 2, and 3 respectively (Table 2). Throughout the trial, VB challenged with Mass41 remained healthy and did not display any notable clinical signs. No gross lesions were observed during necropsies of any birds. Viral RNA (mean 10^5.76^ copy/5µL) was only detected in one bird at the first passage, and was uniquely detected in its tracheal sample.

## 4. Discussion

Following the initial isolation of IBV in the USA during the 1930s, and despite global vaccination of chicken flocks, numerous IBV strains have been detected around the world. Previous studies have suggested that vaccination could be one of the driving factors in IBV genome evolution [7,8]. In the current study, an experimental trial was designed in order to evaluate the evolution of the genetic populations of a vaccine heterologous challenge virus over three passages in unvaccinated and vaccinated SPF chickens. A second experimental trial was designed to control the efficacy of the vaccine against a homologous challenge virus. In the latter trial, viral RNA of the homologous challenge virus was only detected in one vaccinated bird, and only at passage 1; thus, it was concluded that the experimental design was efficient in generating a strong vaccine-induced immune response which blocked homologous challenge virus RNA replication (at least at detectable levels) almost completely. 

Concerning H120-D388 trial, viral RNA was detected in all NVB and VB. After processing NGS data obtained from these samples with VVV [17], a large number of variants were detected in both groups. It was a concern that the one passage in eggs performed for each of these samples in order to optimize NGS and variant analysis could have introduced or eliminated certain variants or modified the percentages of variants in ways that would otherwise not have occurred. However, a comparative analysis of EggP1 D388 with its parental D388 virus resulted in no changes in the viral genetic population at the consensus level and maintenance of 66% of the variants underneath the consensus. The variants selected herein all reached consensus-level percentages within the genetic populations of the different birds, and it was thus concluded that they were unlikely to be due to the single-egg passage procedure. 

Only one variant was selected upon passage in VB to arrive at the consensus level, and this variant was unique to this group; yet, seven were selected upon passage in NVB. Of the seven variants in NVB, four were present in the EggP1 D388 inoculum; thus, by default, VB had the potential to select the same variants as well. However, this did not occur. This would suggest that the virus or viruses carrying the viral RNA of these four variants were eliminated by the immunity induced by vaccination. In both groups, the selection of variants was fast, appearing in the first passage and at the consensus level. Such quick selection has been observed in IBV-Ark type vaccines, in which existing genetic variants were selected and reached the consensus level after only one passage in chickens [20,21].

An interesting observation was the fact that, within a group, the same selected variants were observed in both tracheal and kidney tissues. This may have been a result of the nature of the inoculum being a mixture of tracheal and kidney homogenates from the previous passage; however, it shows that the selected variants did not alter the capability of D388 to replicate in these different tissues.

Three of the eight selected variants were not detected in the viral population of D388 inoculum, meaning that: (i) they appeared during in vivo passages and then were selected at the consensus level; or (ii) they were present in the viral population below the 7% detection threshold. In addition, other variants present in the viral population of D388 inoculum were detected in birds in passage 1 before disappearing, and many variants appeared sporadically in a limited number of birds before disappearing. These observations translate the capacity of the viral population to generate mutations that do not provide a selective advantage and are ultimately eliminated. These results highlight the fact that emerging viruses defined by their consensus sequence are possibly not “new mutants”, and may rather be new viruses selected from an initial population.

Regarding the impact of new amino acid sequences coded by Var2 and Var3 on the functionality of NSP8 and NSP13, respectively, this was assumed to have no effect, as these amino acids were found at high percentages among avian Gammacoronavirus sequences in the NCBI database. It would seem, therefore, that the variant changes are part of a genome molecule of a virus more adapted to the chicken than the parental D388 inoculum, which had undergone previous successive replication in eggs to maintain viral stocks. However, if this is the case, then it is surprising that it was not selected in VB.

Var4 and Var5 induced protein sequence modifications of important size located upstream and in the middle, respectively, of the hypervariable region 1 of the Spike protein. In the present study, virus neutralization studies were conceived in order to assess whether these variants produce viruses that could not be neutralized by antibodies raised against the D388 inoculum; however, these were unsuccessful, as viruses from the NVB and VB were not able to grow on primary chicken kidney cells. As a result, further analysis remains required in order to confirm the immunological impact of the Var4 and Var5 modifications.

Var6 modification led to an increase in the size of the Spike protein by nine amino acids at the C-terminal extremity of the protein; this extension contained a di-lysine motif (KKxx). Di-lysine motifs are known to be endoplasmic reticulum retention signals [22], and the addition of this motif from the Beaudette strain to the vesicular stomatitis virus glycoprotein (G) has been shown to maintain the recombinant G protein in the intermediate compartment between the endoplasmic reticulum and the Golgi [23], which is the budding site of IBV. Most of the published IBV S-genes are predicted to code for the extended form of the Spike protein, and thus this would appear to be a preferred characteristic for the virus in the chicken. Interestingly, although this variant was present in the inoculum, it was not selected in VB.

To conclude, this work revealed that the genetic population of an IBV challenge virus heterologous to an IBV vaccine evolved rapidly under the presented experimental conditions, and evolved completely differently in vaccinated and non-vaccinated chickens. Fewer variants were present in vaccinated birds, which most likely reflects the reduction in diversity of the viral population through reduction of the viral RNA load (approximately ten-fold) as a result of vaccine-induced immunity. It is not known at this point what phenotypic impacts the different variants might have on this strain, or if they would be maintained upon further passage; however, it is clear that populations of viruses did not evolve the same way in birds with different IBV immune statuses. It is important to consider these findings in the context of a field situation, where evolution and pressures occur on a much larger scale, on the order of perhaps 20,000 birds, instead of fifteen vaccinated birds contained under the controlled conditions of the present study. It cannot be known whether the increased number of birds in a field setting would enhance the effects observed in this study or dilute them; however, what is known is that IBV continues to evolve despite the use of live and attenuated vaccines, and that the current study clearly demonstrates the importance of achieving complete vaccine-induced protection in order to avoid genetic drift.

Finally, the present work should be of timely interest considering the current use of human coronavirus vaccines aimed at reducing SARS-CoV 2 infections, the health impact of serious cases of COVID-19, and the related questions on the impact of vaccine-induced immunity on the genetic evolution of this new coronavirus.

## Figures and Tables

**Table 1:** Numbers of seropositive birds and viral RNA RT-qPCR-positive organs in the H120-D388 trial.

	D388 NVB			D388 VB		
	Passage 1	Passage 2	Passage 3	Passage 1	Passage 2	Passage 3
seropositivity (1 d.o.a.)	0/5	0/5	0/5	0/5	0/5	0/5
seropositivity before Passage 1	0/5	0/5	0/5	2/5	2/5	3/5
seropositivity before Passage 2	/	0/5	/	/	2/5	/
seropositivity before Passage 3	/	/	0/5	/	/	5/5
qRT-PCR positive trachea	5/5	5/5	4/5	3/5	5/5	3/5
qRT-PCR positive kidney	5/5	5/5	5/5	5/5	5/5	5/5

**Table 2:** Numbers of seropositive birds and viral RNA RT-qPCR-positive organs in the H120-Mass41 trial.

	Mass41 NVB			Mass41 VB		
	Passage 1	Passage 2	Passage 3	Passage 1	Passage 2	Passage 3
seropositivity (1 d.o.a.)	0/5	0/5	0/5	0/5	0/5	0/5
seropositivity before Passage 1	0/5	0/5	0/5	2/4	3/5	1/4
seropositivity before Passage 2	/	0/5	/	/	4/5	/
seropositivity before Passage 3	/	/	0/5	/	/	3/4
qRT-PCR positive trachea	5/5	5/5	5/5	1/4	0/5	0/4
qRT-PCR positive kidney	2/5	2/5	4/5	0/4	0/5	0/4

## Data Availability

Not applicable.
